# Machine Learning for the Interpretation of Serum Protein and Immunofixation Electrophoresis in Multiple Myeloma: A Scoping Review

**DOI:** 10.3390/diagnostics16142201

**Published:** 2026-07-14

**Authors:** Nuraqila Mohd Murshid, Ahmad Hathim Ahmad Azman, Dian Nasriana Nasuruddin

**Affiliations:** 1 Department of Biochemistry, Faculty of Medicine, National University of Malaysia, Jalan Yaacob Latif, Bandar Tun Razak, Kuala Lumpur 56000, Wilayah Persekutuan Kuala Lumpur, Malaysia; nuraqilamohdmurshid@ukm.edu.my; 2 Department of Medical Education, Faculty of Medicine, National University of Malaysia, Jalan Yaacob Latif, Bandar Tun Razak, Kuala Lumpur 56000, Wilayah Persekutuan Kuala Lumpur, Malaysia; hathimazman@ukm.edu.my; 3 Department of Pathology, Faculty of Medicine, National University of Malaysia, Jalan Yaacob Latif, Bandar Tun Razak, Kuala Lumpur 56000, Wilayah Persekutuan Kuala Lumpur, Malaysia

**Keywords:** multiple myeloma, IFE, electrophoresis, machine learning, diagnosis

## Abstract

**Background**: Serum protein electrophoresis (SPE) and immunofixation electrophoresis (IFE) are essential in diagnosing multiple myeloma (MM). Machine learning (ML) methods have emerged as a potential approach to the automation process of electrophoresis interpretation to improve diagnostic efficiency and improve turnaround time. This scoping review mapped current applications of ML in the diagnosis and classification of MM, with emphasis on automated SPE and IFE interpretation. **Methods**: PubMed, Web of Science, and Scopus databases were searched following PRISMA-ScR guidance. Articles published between January 2015 and April 2025 applying ML to MM diagnosis were included, and findings were synthesised descriptively and thematically. **Results**: Thirteen studies were included, identifying two dominant application domains: ML-based analysis of IFE images and SPE patterns. Deep learning models, particularly convolutional neural networks, achieved expert-level performance in IFE analysis, with peak accuracies reaching 99.82% and F1-scores frequently exceeding 0.90. For SPE interpretation, both deep learning and classical tree-based models demonstrated robust diagnostic yields, achieving Areas Under the Curve (AUC) between 0.90 and 0.99 and overall accuracies ranging from 82.8% to 99.1%. Notably, top-performing models recorded F1-scores up to 0.98, and in some instances, they surpassed the precision of human expert panels. **Conclusions**: Explainable AI methods were increasingly incorporated, though external validation, multimodal integration, explainability, and real-world deployment were limited. ML demonstrates potential to augment SPE and IFE interpretation in MM diagnostics. Future work should focus on multicentre validation, explainability, and clinical integration to support safe implementation.

## 1. Introduction

Multiple myeloma (MM) is a serious and currently incurable blood cancer characterised by the uncontrolled proliferation of plasma cells [1,2]. These abnormal cells often produce a monoclonal immunoglobulin, known as M-protein (historically referred to as paraprotein), which can be found in the blood or urine [3,4]. Early and accurate diagnosis of MM is essential, as it allows for timely treatment that can improve the quality of life and extend the survival of patients [5,6].

Laboratory detection of M-proteins usually involves two main techniques. Serum protein electrophoresis (SPE) serves as the initial screening method to identify abnormal “M-spikes” in the gamma region of the protein profile. While these monoclonal bands most commonly migrate to the gamma region of the protein profile, they can also be present in the alpha-2 or beta fractions, depending on the specific immunoglobulin isotype [7]. Immunofixation electrophoresis (IFE) is used subsequently as a confirmatory test to determine the specific heavy and light chain types of the monoclonal protein [8,9].

Together, SPE and IFE underpin the diagnosis of monoclonal gammopathies such as MM, Monoclonal gammopathy of undetermined significance (MGUS), and Waldenström’s macroglobulinaemia [10,11]. Despite advances in newer diagnostic modalities such as serum-free light chain (FLC) assays and mass spectrometry, current clinical recommendations, including those from the College of American Pathologists (CAP) in their 2021 consensus guideline, continue to endorse SPE with reflex IFE as the initial screening strategy for detecting and characterising monoclonal gammopathies [12,13,14].

The urgency of improving diagnostic pathways is particularly evident in settings facing demographic and infrastructure challenges [6,15]. In Malaysia, for example, MM is the third most common haematological malignancy and primarily affects the elderly [16,17]. Alarmingly, more than half of all cancer cases, including MM, are diagnosed at advanced stages, exposing systemic gaps in early detection and prompt referral. Contributing factors include limited public awareness, low levels of routine screening, and significant disparities in healthcare access between urban and rural areas [18,19,20]. These barriers make it harder for patients to receive fair and timely access to specialised diagnostic services. They underscore the urgent need for scalable, practical solutions that allow even non-specialist healthcare facilities to detect multiple myeloma and other cancers earlier. The findings also show how social, economic, cultural, and emotional challenges intertwine with systemic issues, such as delayed diagnosis, limited access to care, and ultimately worsening patient outcomes [21].

Compounding the issue, the interpretation of SPE and IFE remains a time-intensive and expertise-dependent process [22]. The analysts must manually examine electrophoresis gels or densitometry traces, often interpreting subtle or unclear patterns. This subjectivity introduces variability among operators and increases the likelihood of missing low-concentration monoclonal proteins, especially when results are read by less experienced personnel. The shortage of trained specialists further strains laboratory throughput, particularly in high-volume or underserved settings [23]. To address these bottlenecks, researchers have turned to artificial intelligence (AI) and machine learning (ML) as promising tools for automating the interpretation of electrophoresis data [24]. Early efforts in the 1990s laid the groundwork for using neural networks to identify abnormal patterns in SPE traces [25]. By the early 2000s, several rule-based systems and expert algorithms were introduced, but these initial models were limited by computing power and lacked clinical scalability.

This scoping review aims to chart the current landscape of ML applications for MM diagnostics, with a specific focus on studies targeting automation of SPE and IFE interpretation. We systematically reviewed the literature to identify key applications, diagnostic outcomes, and emerging themes such as explainability, clinical utility, and integration challenges. By mapping both historical and cutting-edge efforts, this review provides a foundation for future development and implementation of AI-assisted diagnostics in MM, particularly in contexts like Malaysia, where diagnostic equity and efficiency are urgent needs.

## 2. Materials and Methods

We conducted this scoping review following the methodological framework proposed by Arksey and O’Malley [26] and refined by the Joanna Briggs Institute (JBI) for scoping reviews. Reporting adheres to the PRISMA-ScR (Preferred Reporting Items for Systematic reviews and Meta-Analyses extension for Scoping Reviews) guidelines [26,27].

The final scoping review protocol has been registered under the Open Science Framework (10.17605/OSF.IO/W6RSP).

### 2.1. Eligibility Criteria

We included peer-reviewed studies, including full articles and brief reports published between January 2015 and April 2025, that employed ML, AI, or advanced computational algorithms to diagnose, detect, or classify MM. Particular emphasis was placed on studies focusing on the interpretation of key laboratory assays relevant to MM diagnosis, such as serum protein electrophoresis (SPE), immunofixation electrophoresis (IFE), and related serum or plasma tests. Studies using computational methods for disease classification or staging were also eligible.

To capture the full scope of the field, studies using image or signal analysis of electrophoresis outputs, natural language processing (NLP) of clinical documentation for MM diagnostic insights, or other data-driven diagnostic frameworks were considered. Both supervised ML models (e.g., neural networks, support vector machines) and expert systems or rule-based algorithms were eligible.

We excluded conference abstracts without complete datasets, review articles, editorials, commentaries, and consensus guidelines to focus on original research evidence. Non-English studies were excluded unless a reliable translation was available. This approach ensured the inclusion of both early foundational and contemporary studies reflecting the evolution of AI-based diagnostics in MM.

### 2.2. Information Sources and Search Strategy

A comprehensive literature search was conducted in PubMed, Web of Science, and Scopus databases covering the period from January 2015 to April 2025.

The search strategy was developed in collaboration with a medical librarian, and combined keywords and subject headings related to multiple myeloma (and plasma cell neoplasms), including terms for machine learning, artificial intelligence, neural networks, deep learning, computer-assisted diagnosis, and specific test terms like electrophoresis and immunofixation. Broad terminology was deliberately utilised for these diagnostic modalities to maximise search sensitivity; for example, omitting specific “serum” or “urine” modifiers ensured the capture of all relevant studies without artificially restricting the literature yield. In addition, the reference lists of included articles were manually screened to identify further relevant studies not captured by the database searches.

### 2.3. Research Strategies and Operator Keywords

The review incorporates article findings sourced from the Scopus, PubMed, and Web of Science (WoS) databases. The search strategy focuses on the following phrases and Boolean operators, as in Table 1.

### 2.4. Study Selection

**Figure 1:**
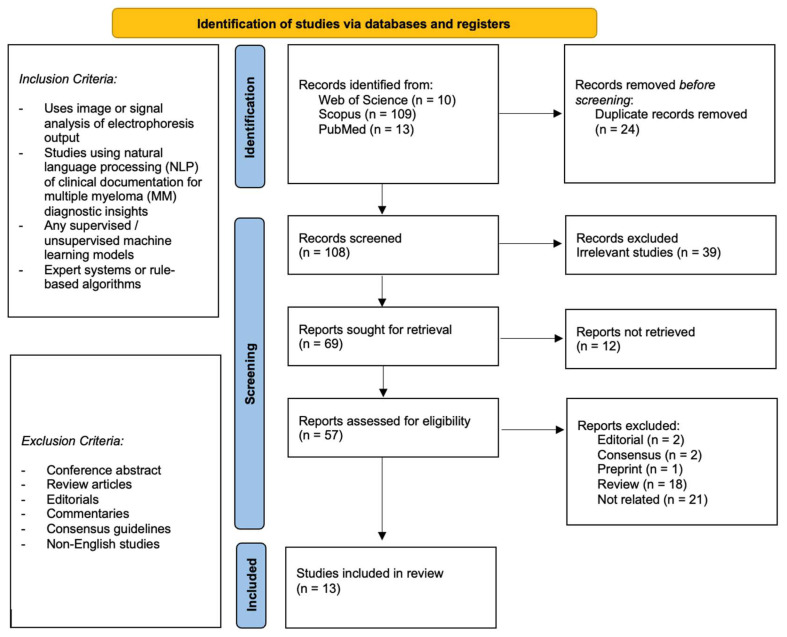
PRISMA 2020 Flow diagram for application of machine learning in classification and diagnosis of multiple myeloma.

All retrieved records were imported into a reference management tool, EndNote 20, and duplicates were removed. Two reviewers independently screened the titles and abstracts against the eligibility criteria. Any record clearly not related to ML in MM diagnosis (e.g., studies on MM treatment, papers on general AI not applied to MM, etc.) was excluded at this stage. Full-text articles were obtained for all remaining citations, and each was assessed in detail for eligibility. Any disagreements between reviewers were resolved through discussion or consultation with a third reviewer. Exclusion reasons at the full-text stage were recorded. The study selection process is illustrated in a PRISMA flow diagram (Figure 1). In summary, out of 132 records identified (after duplicates were removed), 57 full-text reports were assessed, and 13 studies met all criteria and were included in the review.

### 2.5. Data Extraction and Charting

We developed a data extraction form based on JBI guidance to capture key study information. Both authors extracted data elements including: bibliographic details (author, year, country), the diagnostic context (type of test or data used, e.g., SPE, IFE, clinical records, etc.), sample characteristics (number of samples or images, data source), the machine learning approach (algorithm/model type, architecture, and any software or tool used), the objective of the model (e.g., detect any M-spike, classify isotype, predict stage), and outcomes/results (performance metrics like accuracy, sensitivity/specificity, AUC, error rates; comparisons to human readers; and any qualitative outcomes like interpretability or workflow impact). We also documented any mention of specific themes such as use of explainable AI techniques, integration into clinical workflow, multi-modal data integration, or efforts to ensure generalisability (external validation).

Data extraction was performed independently by two reviewers, who subsequently compared their findings to ensure consistency and completeness. Disagreements were initially addressed through mutual discussion to achieve consensus; any unresolved discrepancies were adjudicated by a third expert reviewer. The extracted data were subsequently tabulated and categorised. In accordance with standard scoping review methodology, a formal quality appraisal was not conducted. However, key quality indicators, including the presence of external validation, were documented to gauge methodological rigour.

### 2.6. Synthesis of Results

We synthesised the findings descriptively, employing a thematic approach aligned with our review objectives. Since a large subset of included studies fell into two distinct modalities, i.e., IFE image analysis and SPE pattern analysis, results and discussion were organised accordingly. Within each group, we further explored six common themes: (1) diagnostic performance of the ML models, (2) explainability and transparency of the algorithms (e.g., use of XAI methods), (3) clinical integration and workflow considerations, (4) model architecture and computational requirements, (5) presence of multimodal integration (combining multiple data sources), and (6) data diversity and the generalisability of the models. We present summary tables for the IFE- and SPE-focused studies in Table 2 and Table 3, respectively. This is followed by a narrative synthesis structured around these themes. This thematic evidence-mapping approach enables us to highlight how each included study contributes to different facets of the overall research question and to identify concentrations or gaps in the evidence.

## 3. Results and Discussion

### 3.1. Study Characteristics

A total of 13 studies met the inclusion criteria and were included in this review. The publication years ranged from early 2015 (initial exploratory efforts to apply neural networks to electrophoresis) to 2025, reflecting a progressive increase in interest paralleling improvements in computing and data availability. Most studies were observational, involving retrospective analyses of stored laboratory data or images; no interventional trials were identified.

We observed that the included studies predominantly focused on utilising ML for the laboratory diagnosis of MM by analysing protein electrophoresis results. The characteristics of the 13 included studies were divided into two main categories based on the targeted diagnostic modality, which were immunofixation electrophoresis (IFE, *n* = 5) and serum protein electrophoresis (SPE, *n* = 8). A limited number of studies incorporated both IFE and SPE data; creating a separate multimodal category was deemed impractical due to the small sample size. Consequently, studies exhibiting overlapping modalities were categorised into either the IFE- or SPE-focused groups according to their primary analytical focus or the dominant dataset utilised for model evaluation. A complete extraction of the objectives, data sources, AI techniques, and outcomes for all included studies is provided in Table 2 and Table 3.

#### 3.1.1. IFE-Focused Studies

These investigations, shown in Table 2 (*n* = 5 studies), applied ML to immunofixation electrophoresis images. Typically, they aimed to automatically identify monoclonal bands on IFE gels and determine their immunoglobulin heavy- and light-chain types (e.g., IgG kappa, IgA lambda), essentially replicating manual interpretation of IFE results. The data for these studies consisted of digitised gel images or scans, often labelled by experts for training. Deep learning (especially CNNs) was the dominant approach in this category, given the image-based nature of IFE.

#### 3.1.2. SPE-Focused Studies

These studies in Table 3 (*n* = 8 studies) utilised ML on serum protein electrophoresis patterns or signals. Objectives varied from simply detecting an M-spike (paraprotein peak) in an SPE densitometry curve (yes/no screening) to quantifying M-protein concentration or classifying entire electrophoretic patterns. Input data included digital electrophoretic curves (from gel or capillary systems) or even raw image plots of SPE tracings. A wider range of ML techniques was seen here—from early artificial neural networks (ANNs) and expert systems to modern deep learning models and custom algorithmic analyses.

### 3.2. Thematic Synthesis of Included Studies

The included studies demonstrate a consistently high diagnostic performance for ML models across both IFE and SPE modalities. IFE-focused studies primarily target automated pattern recognition and the localisation of monoclonal bands on IFE gels, including their immunoglobulin heavy- and light-chain types. Deep learning, particularly convolutional neural networks, dominates this domain due to the image-based nature of IFE data. For example, Wei, Yang, Zhang, Liao, Sheng, Zhou, Wu and Du [28] showed significant improvements in F1 scores with deep collocative learning, while Hu, Xu, Jiang, Cheng, Tao, Liu, Jian, Li and Wang [32] reported an accuracy of 99.82% in classifying eight basic IFE patterns. Thiemann, Klitzke, Martinetz, Gruening, Käster, Barth, Kramer and Martinetz [30] zero false positives and one false negative on a large set of confident cases, indicating robustness classification for clinical deployment.

In addition to AUC, research on IFE repeatedly shows that machine-learning models excel in other critical diagnostic parameters, such as accuracy, recall, precision, and F1-score. These measurements provide a comprehensive assessment of model reliability, demonstrating not only the algorithms’ ability to differentiate between classes but also their confidence in identifying true positives and minimising false positives. This tendency is consistently seen across published works. Vilarinho Filho, Couto and Souza [31] reported F1-scores exceeding 0.90 in the analysis of challenging IFE images, whilst Hu, Xu, Jiang, Cheng, Tao, Liu, Jian, Li and Wang [32] achieved near-perfect accuracy and specificity, demonstrating performance akin to that of seasoned laboratory professionals. Thiemann, Klitzke, Martinetz, Gruening, Käster, Barth, Kramer and Martinetz [30] similarly reported high accuracy with minimal false positives, underscoring the reliability of automated interpretation in practical datasets. Explainability-driven models, including the collocative learning approach proposed by Wei, Yang, Zhang, Liao, Sheng, Zhou, Wu and Du [28], have achieved significant performance gains over traditional CNNs. These results collectively demonstrate that ML-based IFE interpretation is both reliable and consistently resilient across several diagnostic dimensions.

As we observed that across both modalities, the primary objective is to replicate and, in some cases, exceed human expert performance in detecting and characterising monoclonal proteins, thereby reducing subjectivity and improving consistency.

The collective finding of SPE-focused studies consistently showed that ML models can achieve high diagnostic performance in detecting and quantifying monoclonal proteins. Chabrun, Dieu, Ferre, Gaillard, Mery, Chao de la Barca, Taisne, Urbanski, Reynier and Mirebeau-Prunier [33] developed a multimodal deep learning architecture and achieved an AUC of >0.99 for M-spike proteins, while Lee, Jeong, Jeon, Song and Park [38] utilised DenseNet-121, a deep learning algorithm modelled on densitograms. It achieved AUC ranging from 0.873 to 0.989, outperforming or even matching expert-level interpretations. Studies that focus on classical ML models often converge on tree-based models, as done by Cherkaoui, Penickova and Cotton [35], Elfert, Kaminski, Matek, Hoermann, Axelsen, Marr and Piehler [24], and Sopasakis, Nilsson, Askenmo, Nyholm, Mattsson Hultén and Rotter Sopasakis [37], who all achieved AUC > 0.9 with different tree-based models such as Random Forest, Gradient Boosting, and Extra Trees. Beyond AUC, SPE-focused studies consistently reported strong performance across other key diagnostic metrics such as accuracy, recall, precision, and F1-score, further reinforcing the robustness of ML approaches for M-protein detection and quantification. Elfert, Kaminski, Matek, Hoermann, Axelsen, Marr and Piehler [24] demonstrated that a Random Forest classifier could achieve 99.1% accuracy, 89.9% sensitivity, 99.8% specificity, 96.9% precision, and an F1-score of 0.93, surpassing the performance of ten laboratory experts, particularly in precision and overall diagnostic balance. Similarly, Sopasakis, Nilsson, Askenmo, Nyholm, Mattsson Hultén and Rotter Sopasakis [37] reported that ensemble tree-based methods such as Extra Trees and Random Forest reached accuracies of 96–98% and F1-scores around 0.98, supported by SHAP-validated feature contributions emphasising physiologically relevant electrophoretic regions. Classical ML models with smaller datasets showed more modest but still meaningful performance, while Cherkaoui, Penickova and Cotton [35] reported ∼85% sensitivity and AUROC 0.91 for early monoclonality detection based on β-region anomalies. Deep learning approaches such as DenseNet-121 in Lee, Jeong, Jeon, Song and Park [38] maintained 85–97% accuracy across diagnostic classes depending on the image modality (gel vs. densitogram), indicating strong performance even in multi-class settings. Collectively, these findings show that SPE-based ML models consistently deliver high recall for clinically relevant abnormalities, extremely high precision for ruling in monoclonal components, and well-balanced F1-scores, making them suitable for both screening and confirmatory diagnostic workflows.

### 3.3. Explainability and Model Transparency

The importance of interpretability in diagnostic AI is increasingly recognised, especially in critical applications such as MM diagnosis. Several studies explicitly incorporate or propose explainability techniques. Wei, Yang, Zhang, Liao, Sheng, Zhou, Wu and Du [28] employed Coached Attention Gates and Grad-CAM for their IFE model, aiming to replicate human logic. Similarly, Hu, Xu, Jiang, Cheng, Tao, Liu, Jian, Li and Wang [32] developed an ensemble CNN with Score-CAM explainability, emphasising the importance of visibility into model behaviour for building clinician trust. In the SPE domain, Chabrun, Dieu, Ferre, Gaillard, Mery, Chao de la Barca, Taisne, Urbanski, Reynier and Mirebeau-Prunier [33] developed an explainable multi-module AI system (SPECTR) by clearly delegating roles for each model within the decision pipeline. In contrast, the rule-based densitometry boundary detection developed by Clavijo, Ryan, Xu and Singh [34] provided clear interpretability through deterministic peak-boundary logic, allowing identification of machine overestimates. While Sopasakis, Nilsson, Askenmo, Nyholm, Mattsson Hultén and Rotter Sopasakis [37] further advanced interpretability by using SHAP to quantify the importance of gamma and beta regions for M-protein detection, providing valuable insights into model decisions.

### 3.4. Clinical Integration and Workflow Considerations

Many studies identify clear potential for ML models to function as decision-support tools to enhance clinical workflows and integration, although real-world implementation remains a key future goal. Thiemann, Klitzke, Martinetz, Gruening, Käster, Barth, Kramer and Martinetz [30] focused on clinical deployment, aiming for AI-assisted diagnostics. Chen et al. proposed open-source tools and web services for real-time screening, especially relevant for low-resource settings. Malek, Wang, Tatsuoka, Cullen, Madabhushi and Driscoll [36] explored point-of-care prediction of M-spike values using electronic health record data, demonstrating potential for rapid disease monitoring and faster decision-making, thereby circumventing lengthy lab turnaround times. Chabrun, Dieu, Ferre, Gaillard, Mery, Chao de la Barca, Taisne, Urbanski, Reynier and Mirebeau-Prunier [33] explicitly addressed the potential integration of SPECTR into existing Laboratory Information Systems, signalling a potential in AI-enhanced workflows.

Other studies such as Lee, Jeong, Jeon, Song and Park [38] highlighted the need for integrating clinical information (e.g., disease history, medication history, examination findings) and complementary laboratory findings (e.g., blood parameters, previous electrophoresis images) with these models, to avoid siloed decision-making and to fully leverage the diagnostic capabilities of these ML algorithms. Despite the evident potential, most integration efforts remain conceptual or in early development stages.

A lack in multimodal fusion is also a point of consideration, with most studies relying on a single data source, either raw SPE curves, isolated clinical chemistry, or raw CZE signals [33,35,37]. Given the evident performance of single-modality models, future models should incorporate electrophoretic patterns alongside laboratory biomarkers, radiological features, and even genomic data to enhance classification performance, especially for underrepresented isotype classification and small M-protein detection.

### 3.5. Data Diversity and Generalisability

The greatest challenge highlighted across the studies was the limited diversity and generalisability of the datasets. Data diversity and generalisability are critical themes influencing the real-world utility of these models. Dataset sizes vary significantly, ranging from 127 samples to nearly 160,000 SPE samples [33]. Many studies originated from single institutions using homogenous laboratory equipment, raising concerns regarding potential single-centre bias and overfitting.

However, some studies addressed this issue through external validation, such as studies by Chabrun, Dieu, Ferre, Gaillard, Mery, Chao de la Barca, Taisne, Urbanski, Reynier and Mirebeau-Prunier [33], which validated SPECTR on 70,362 external test SPEs, and Chen, Orom, Hay, Waters, Schofield, Li and Kiviniemi [18], which included 1321 external test samples. Several “Future Directions” sections explicitly call for multicentre validation and robustness against variable staining or noise conditions [28,31]. The exploration of “domain-shift” [32] acknowledges the challenges in generalising models trained on one laboratory’s data to another. Rare monoclonal patterns were consistently underrepresented, creating potential blind spots in real-world deployment, which underscores the need for larger, more diverse datasets capturing the full spectrum of MM presentations to achieve broadly generalisable and clinically safe ML diagnostic tools. This can be achieved through multi-institutional collaborations and data-sharing initiatives.

The current evidence highlights several significant strengths and innovations in applying machine learning (ML) to multiple myeloma (MM) diagnostics. First and foremost, among these is the consistency of ML-based models in achieving high diagnostic accuracy, with many models reaching expert-level or even superior performance in M-spike detection, quantification, and pattern classification for both serum protein electrophoresis (SPE) and immunofixation electrophoresis (IFE) [24,32,33,37]. Equally important is the enhanced consistency of ML-driven models: by standardising the analysis process, these models reduce inter-operator variability and improve reproducibility compared to manual readings and interpretations [33,37,38]. Together, these attributes demonstrate that ML can provide not only highly accurate but also more reliable diagnostic interpretations. Another major advantage of ML introduced in this domain is the potential for automation and increased efficiency. Automated ML interpretation of electrophoretic patterns can decrease reliance on manual interpretations by time-intensive, expert-dependent manual analysis, thereby significantly increasing laboratory throughput and shortening the turnaround time for results [36]. Automation of routine analyses enables higher throughput and faster reporting, allowing for more timely clinical decision-making and improving patient management.

Another area of innovation that researchers are proactively exploring is incorporating explainable AI (XAI) techniques into ML diagnostic tools to address the “black-box” concern. As researchers delve more into complex algorithms from classical machine learning to deep learning architectures, these different types of explainable AI, such as attention-based visualisation methods [38], SHAP-based feature attribution [37], or even structured multi-module architectures [33], further provide meaningful insights and offer inherent transparency by understanding the systematic biases and decision workflow. This emphasis on interpretability allows clinicians to understand the rationale behind the algorithm’s conclusions, fostering greater trust and easing the path toward clinical adoption [28,32,37]. By clarifying how models weigh different features or highlighting key indicators in the decision-making, XAI-enhanced systems ensure that AI-driven insights are transparent and align algorithmic reasoning with established clinical logic, thereby encouraging their acceptance in practice for both practitioners and patients.

Finally, ML expands MM diagnostics through early detection and predictive monitoring capabilities. Some systems are able to detect subtle abnormalities indicative of monoclonal gammopathy at an earlier stage, which could trigger reflexive confirmatory tests or closer surveillance sooner than traditional approaches [35]. Similarly, ML-driven tools provide rapid (even real-time) monitoring of disease biomarkers such as the M-spike, allowing for quicker treatment adjustments and more proactive patient management [36]. These predictive capabilities create opportunities for dynamic treatment adjustment, more responsive patient care, and illustrate how ML can shift diagnostics from a reactive process to a more proactive, continuous form of patient care.

## 4. Risk and Bias Assessment

To evaluate the methodological quality and applicability of the included studies, a risk of bias assessment was conducted according to the Prediction model Risk Of Bias Assessment Tool (PROBAST+AI) [40]. As an extension of the original PROBAST guideline tailored specifically for artificial intelligence and machine learning applications, this tool provides a structured approach to identifying potential methodological flaws in model development and validation. The assessment systematically evaluates four key domains to ensure that common AI-specific pitfalls, such as data leakage and overfitting, are properly scrutinised. Domain 1 (participants and data sources) focuses on the data used for model development and training. Domain 2 (predictors) covers the definition and measurement of the predictors utilised. Domain 3 (outcomes) evaluates the measurement of the predicted outcomes in a manner similar to Domain 2. Lastly, Domain 4 (analysis) comprises questions that support both the quality assessment of model development and the risk of bias assessment for model evaluation. In this review, all included studies involved both model development and evaluation, with the exception of study [34], which was analytical and system-based in nature. Consequently, the assessment criteria for both development and evaluation phases were applied to all eligible studies.

### 4.1. Assessment of Model Development

Across the 13 evaluated studies, performance regarding participants and data sources was generally strong, with all studies utilising appropriate data sources and study designs, although three studies [34,35,36] relied on cohorts with questionable representativeness. Predictors and outcomes were consistently well-defined and uniformly processed across the board. Conversely, a universal vulnerability was observed regarding blinding protocols; it was unclear in all studies whether predictor assessments were blinded to outcomes, and none explicitly confirmed that outcome determination was blinded to predictor information. Finally, the analysis domain exhibited the greatest variability. While continuous variables were handled appropriately and overfitting mitigation was broadly adopted, four studies [31,35,36,39] operated on limited sample sizes relative to model complexity.

The assessment identified significant analytical weaknesses regarding sample size adequacy and class imbalance recalibration. Nearly 40% of the evaluated studies operated on sample sizes that were insufficient for the mathematical complexity of their respective machine learning models. Small datasets in highly dimensional AI models substantially increase the likelihood that the model will capture statistical noise instead of meaningful underlying patterns. Coupled with this is the inconsistent management of data imbalance. In healthcare, predicted outcomes are naturally skewed. Over half of the studies provided unclear information on whether this imbalance was recalibrated. Failing to address class imbalance often results in algorithms that appear highly accurate overall but perform dangerously poorly on the minority class [41]. Moving forward, the clinical applicability of these models will depend heavily on researchers adopting rigorous sampling strategies and clearly reporting techniques such as the Synthetic Minority Over-sampling Technique (SMOTE) or algorithmic class weighting [42] (Table S1).

### 4.2. Assessment of Model Evaluation

The risk of bias assessment during the model evaluation phase maps the critical transition from algorithmic training in silico to validation testing. Performance across the initial structural domains closely mirrored the development phase, maintaining strong data provenance alongside the persistent, universal ambiguity surrounding data blinding protocols. Clavijo (2020) [34] was excluded due to its purely analytical nature, leaving 12 studies for evaluation. Performance across the first three domains closely mirrored the development phase; studies universally utilised appropriate data sources, study designs, and interval timings, while defining predictors and outcomes with high standardisation. However, dataset representativeness remained a concern for the same two studies [31,35], and the critical lack of blinding protocols persisted universally across both predictor and outcome assessments. The analysis domain revealed distinct methodological shifts during the evaluation phase. Notably, all 12 evaluated studies achieved a reasonable sample size for evaluation, universally evaluated performance appropriately (Question 4.7), and successfully replicated resampling techniques in all applicable instances (Question 4.6). Conversely, the handling of continuous and categorical variables deteriorated during validation, with four studies [31,35,36,39] failing to manage them appropriately. Furthermore, the lack of transparency regarding data imbalance recalibration persisted, with only four studies explicitly confirming that adjustments were made during the evaluation phase.

The evaluation phase assessment underscores a consistent foundational strength across the reviewed literature, particularly in how data sources are selected and variables are defined. However, the universal absence of blinding during model evaluation remains a profound methodological limitation. While the retrospective nature of electronic health and educational records makes traditional blinding difficult, it is crucial during the validation phase to ensure that those curating the test datasets or interpreting the model’s predictions are not influenced by prior knowledge of the actual outcomes [43].

The results highlight a strong adherence to advanced validation protocols, with near-universal replication of resampling techniques such as cross-validation or bootstrapping, alongside comprehensive performance evaluations. This indicates that researchers are actively employing rigorous standards to verify model stability. Nevertheless, these robust testing frameworks exist alongside a persistent failure to adequately report or address data imbalance. If a test dataset remains heavily skewed towards a majority class, standard evaluation metrics like overall accuracy can appear misleadingly high, masking the model’s potential failure to predict rare but critical outcomes. For these AI models to be safely translated into active healthcare or educational settings, researchers must pair their strong resampling methodologies with transparent imbalance recalibration techniques and report minority-class specific metrics such as precision, recall, and the F1-score [44] (Table S2).

### 4.3. Overall Assessment

The publication bias toward studies reporting favourable ML performance should be acknowledged. Studies with negative or inconclusive results are less likely to be published, which may lead to an overly optimistic portrayal of ML diagnostic accuracy across the literature. This selective reporting warrants caution when interpreting the collective performance metrics presented in this review (Table S3).

## 5. Limitations and Gaps

Despite recent advances, our review identified several significant limitations across the studies. As summarised in Table 4, these gaps can be broadly categorised into four main themes: standardisation and generalisation, explainability and trust, multimodal data fusion, and clinical integration and workflow. Firstly, dataset size and origin remain a primary concern. While a few models were trained on large datasets, many relied on relatively small, single-centre retrospective cohorts, which limits the generalisability of their findings [45]. Alongside this, there is a lack of external validation. Only a few studies tested their algorithms on independent datasets from different laboratories or patient populations. This absence of multi-centre validation makes it difficult to judge how robust these models would be in diverse real-world settings [46]. Furthermore, there is a variable comparison with human experts [47]. While some studies directly benchmarked their model’s performance against experienced clinicians, others did not or only made indirect comparisons against junior staff or historical norms [48]. Clear performance benchmarks against consensus expert interpretations were often lacking [49]. In one study, an automated system consistently measured monoclonal protein concentrations higher than those of the experts, showing a positive bias in the automated quantification [34]. These inconsistencies underscore the necessity for thorough calibration of algorithms in relation to expert judgement.

Another critical gap observed is the prevalence of “black-box” models. Despite increasing interest in explainable AI, the inherent complexity and opacity of many deep learning methodologies pose challenges for regulatory validation and clinical acceptance. Notably, none of the studies identified in this review applied multimodal explainable AI techniques, which may increase the accuracy and expressiveness of the explanations [50]. In the absence of interpretable reasoning or transparency, laboratory personnel may hesitate to trust these AI outputs [51,52].

The current scope of studies tends to focus heavily on specific assays in isolation, primarily SPE and IFE. There has been less exploration of combining these results with other key diagnostic modalities. Merging different data sources, such as combining numerical capillary electrophoresis (CE) data with gel image analysis, would suggest enhancing classification accuracy [37]. Expanding this to incorporate serum-free light-chain assays, clinical text, imaging findings, or genomic and histological data could provide a more holistic diagnostic picture for multiple myeloma [29,36], indicating that a multimodal approach is a highly promising yet underdeveloped area.

Finally, the incorporation of these algorithms into the clinical laboratory workflow remains limited. Most studies were retrospective analyses of historical data; prospective trials or real-world implementation studies are largely missing. As a result, the purported clinical utility of these machine learning tools remains theoretical until they are evaluated in live clinical workflows. Most authors discuss scaling to larger datasets, integrating SPEP-IFE matching logic, and incorporating their tools into Laboratory Information Systems (LIS) as future goals rather than describing successful integration to date [33]. Establishing clinical utility will also require determining safe probability cutoffs to streamline workflows, such as deciding when to safely skip IFE confirmation [35]. In summary, limited data diversity, inconsistent human benchmarks, opaque algorithms, narrow modality focus, and a lack of real-world workflow integration represent the critical gaps that future research must address.

## 6. Clinical Relevance: Augmentation, Assistance, or Replacement?

Based on current evidence, ML models are well-positioned to augment and assist human interpretation in MM diagnosis, with the potential to replace humans in highly specific, well-defined tasks. At present, ML systems serve as powerful aids that can enhance expert capabilities. They are adept at screening large volumes of samples rapidly, flagging suspicious patterns, and providing precise quantitative analyses [53]. For example, the SPECTR deep learning system developed by Chabrun, Dieu, Ferre, Gaillard, Mery, Chao de la Barca, Taisne, Urbanski, Reynier and Mirebeau-Prunier [33] achieved expert-level interpretation of serum protein electrophoresis, accurately detecting and quantifying M-spikes and other abnormalities. Notably, SPECTR’s agreement with human experts was as good as, or even slightly better than, the agreement among different experts. This performance illustrates how automation can enhance throughput, consistency, and sensitivity in standard myeloma diagnostics. By handling the labour-intensive initial analysis and measurements with high precision, these tools free up specialists to focus on complex cases and verification [54].

Machine learning models may function as intelligent assistants that facilitate decision-making [55]. They provide preliminary classifications and highlight subtle features that a busy technologist or less experienced clinician might overlook. For instance, some deep learning approaches for immunofixation electrophoresis now achieve human-level pattern recognition and do so with built-in explainability, marking specific bands or regions to show why a sample is flagged [32]. This kind of assistance is especially valuable in settings with limited access to skilled haematopathologists or when the workload is high. Furthermore, AI systems can be trained to recognise when they are unsure, effectively saying “this case is inconclusive,” so that ambiguous findings are set aside for manual review [56]. Thiemann, Klitzke, Martinetz, Gruening, Käster, Barth, Kramer and Martinetz [30] demonstrated this approach by using a confidence threshold: their automated IFE assessment system could handle roughly 75% of cases autonomously and deferred about 25% as “inconclusive” for expert interpretation. Importantly, in the subset of cases the model called conclusively, it made virtually no false-positive errors and only a single false-negative, indicating that it can safely assist by triaging straightforward cases to automation while reserving the difficult ones for human input. This interaction between AI and experts makes sure that overall diagnostic efficiency is up without affecting accuracy in the challenging situations [57].

In the future, it is likely that for some highly specified tasks, machine learning systems might totally displace human readers [58]. The high accuracies reported for specific objectives, such as detecting an M-spike or classifying a typical monoclonal pattern, suggest that automation can match expert performance in those domains. We assess that full replacement of human expertise is not yet realistic except in highly controlled scenarios. There are several reasons for caution [59]. Clinicians have significant responsibility in diagnosing patients, and existing rules and liability concerns mean that an expert must make the final decision. Furthermore, edge cases and unusual manifestations of multiple myeloma necessitate the discerning judgement derived from medical training and experience [6].

The earlier example of positive bias in automated quantification underscores how an uncorrected algorithm might systematically drift in its readings, potentially misleading management if not caught by a human [34]. Therefore, even as automation increasingly takes over repetitive tasks, humans are still needed for complex or borderline cases, leveraging our higher-order thinking skills rather than relying on the machine’s predictions. In summary, today’s ML tools for myeloma function best as adjuncts that augment and assist laboratory experts. With further improvements, they might reliably replace humans in certain routine interpretations, but at present, they should be integrated as supportive elements of the diagnostic process rather than standalone replacements for expert judgement. Ultimately, the goal is to improve diagnostic accuracy, speed, and reproducibility, thereby positively impacting patient outcomes, especially in regions facing diagnostic delays and disparities.

## 7. Future Directions

To fully realise the potential of ML in MM diagnostics, focused efforts are needed in several key areas:

### 7.1. Larger, Multi-Centred, Multi-Platform Datasets and Open Benchmarking

There is an urgent need to develop extensive, diverse, standardised datasets collected from multiple institutions and using various laboratory platforms. Such a dataset will enable robust external validation and reduce overfitting to single-centred patterns, ensuring models generalise effectively across diverse clinical environments. Establishing open benchmarking datasets for SPE, IFE, and potentially SFLC data in MM would provide standardised benchmarks for evaluating and comparing emerging ML algorithms.

### 7.2. Development of Explainable and Clinically Interpretable AI Models

Future research should prioritise advanced explainability techniques that go beyond accuracy to provide transparent, clinician-friendly rationales for model decisions. This is paramount for building trust, facilitating regulatory approval, and enabling lab accreditation, moving beyond “black-box” perceptions towards alignment with clinical reasoning.

### 7.3. Integration of Multiple Modality in Data, Models, and Explainable AI

Future efforts should progress beyond siloed diagnostic pipelines by integrating multimodal data, models, and explainability frameworks. Although single-modality approaches such as SPE curves, IFE images, or structured laboratory data perform well independently, incorporating additional modalities such as bone marrow findings, imaging results, genomic profiles, serum biomarkers, and longitudinal clinical parameters could provide a more comprehensive foundation for MM diagnosis, classification, and personalised treatment prediction. At the model level, multimodal fusion architectures that combine CNNs, transformers, and ensemble methods offer the potential to jointly learn from heterogeneous data types and capture complementary biological and electrophoretic patterns that isolated models may overlook. Correspondingly, explainable AI must evolve to provide clinically coherent multimodal interpretations, moving beyond single-source CAM or SHAP maps to frameworks capable of showing how different data streams collectively inform a prediction. Together, multimodal data integration, multimodal modelling, and multimodal explainability represent critical next steps toward more holistic, accurate, and clinically trustworthy AI systems for MM diagnostics.

### 7.4. Prospective Trials, Real-World Implementation Studies, and Cost-Effectiveness Analyses

Transitioning from retrospective proof-of-concept studies to prospective, interventional trials is essential. These studies must evaluate ML models in real-world clinical workflows, assessing their impact on patient management, diagnostic turnaround times, and overall healthcare costs. Cost-effectiveness analyses will be vital for justifying widespread adoption.

### 7.5. Standardisation of Reporting and Adherence to AI Reporting Guidelines

To ensure reproducibility and facilitate meta-analyses, there is a critical need for standardised reporting of ML studies in MM diagnostics. Adherence to established guidelines such as Transparent Reporting of a Multivariable Prediction Model for Individual Prognosis or Diagnosis (TRIPOD-AI) and Consolidated Standards of Reporting Trials (CONSORT-AI) will improve the quality, rigour, and comparability of research in this rapidly evolving field.

### 7.6. Addressing Practical Challenges of Deployment

Future research should address the practical challenges associated with real-world deployment, including regulatory pathways, LIS integration, user training, model maintenance, and handling variations in laboratory protocols and instrument calibrations.

## 8. Conclusions

This scoping review highlights the transformative potential of machine learning (ML) in automating the interpretation of serum protein electrophoresis (SPE) and immunofixation electrophoresis (IFE). These computational tools have demonstrated expert-level accuracy and offer a scalable solution to diagnostic bottlenecks in resource-limited settings. Automating detection could significantly enhance diagnostic capacity in low-resource hospitals and facilitate earlier intervention for multiple myeloma. However, the clinical translation of these models remains hindered by a prevalence of retrospective studies, a lack of multi-institutional data sources, and a lack of robust external validation. Future research must therefore prioritise multicentre collaborations and the development of Explainable AI (XAI) architectures to ensure these systems are not only accurate but also transparent and trustworthy enough for routine clinical integration.

## Figures and Tables

**Table 1:** Keyword selection.

Keywords	Operators
Disease	(“multiple myeloma” OR “plasma cell neoplasm*”OR “monoclonal gammopath*” OR “monoclonal protein*” OR “M-protein” OR “paraprotein”)
Diagnostic Modality	(“serum protein electrophoresis” OR “SPE” OR “protein electrophoresis” OR “immunofixation electrophoresis” OR “IFE” OR “immunotyping” OR “capillary electrophoresis” OR “gel electrophoresis”)
Machine Learning/AI	(“machine learning” OR “artificial intelligence” OR “deep learning” OR “neural network*” OR “convolutional neural network*” OR “CNN” OR “support vector machine*” OR “SVM” OR “computer-assisted diagnosis” OR “pattern recognition” OR “automated interpretation” OR “image analysis” OR “signal analysis”)

Note. IFE: Immunofixation Electrophoresis, SPE: Serum Electrophoresis, CNN: Convoluted Neural Network, SVM: Support Vector Machine.

**Table 2:** PRISMA-ScR data extraction table for IFE and related ML studies.

Authors	Objective	Method Technique and AI	Dataset (*n*)	Results
Wei, et al. [28]	Automate IFE pattern recognition and localise collocated bands (IgG, IgA, IgM, *κ*, *λ*)	Deep Collocative Learning (DCL) + Coached Attention Gates + Grad-CAM	4352 images IFE	F1 improved vs. ResNet18; IoU ↑ 741.3%, PrecisionR ↑ 118.4%, RecallR ↑ 725.1%
An, et al. [29]	Two-stage cascade: detect M-protein presence, then isotype classification	Cascade Deep Learning (DCL + Recurrent Attention Model)	22,470 images IFE	Accuracy ↑ 0.94–1.0%, F1 ↑ 5.32–6.10% vs. baselines
Thiemann, et al. [30]	Automated 10-class immunofixation classification	ResNet18 CNN with confidence thresholding	>4000 IFE images	0 false positives, 1 false negative on 3000 confident cases; 25% flagged as “inconclusive”
Vilarinho Filho, et al. [31]	Compare CNN vs. handcrafted feature NN for IFE classification	VGGNet, DenseNet CNNs, and dense NN with handcrafted feature vector	Balanced dataset (hundreds)	CNN F1 = 0.91; Dense NN F1 = 0.96, Precision = 0.96
Hu, et al. [32]	Develop explainable, generalizable AI for 8 basic IFE patterns	Ensemble of 3 CNNs +Score-CAM explainability	12,703 IFE images	Accuracy = 99.82%, Sensitivity = 93.17%, Specificity = 99.93%

Note. IFE: Immunofixation Electrophoresis, CNN: Convoluted Neural Network, NN: Neural Network, IoU: Intersection over Union.

**Table 3:** PRISMA-ScR data extraction table for SPE and related ML Studies.

Authors	Objective	Method and AI Technique	Dataset (*n*)	Results
Chabrun, et al. [33]	Develop SPECTR: expert-level AI for SPE interpretation.	Multi-network DL (Fractioning, Class, Peak, Hemolysis) + Rule-based Integrator.	230,331 total SPEs	Cohen’s κ = 0.632 vs. experts; 72% perfect concordance with human consensus.
Clavijo, et al. [34]	Compare automated vs. manual M-spike quantification.	Densitometry vs. visual quantitation (Statistical analysis).	300 SPE gels	r = 0.997 correlation, but automated reading showed +0.29 g/dL positive bias.
Cherkaoui, et al. [35]	Predict monoclonal protein when β-globulin is elevated.	Five ML classifiers (Random Forest selected).	309 patient samples	Random Forest achieved 85% sensitivity and AUROC 0.91.
Elfert, Kaminski, Matek, Hoermann, Axelsen, Marr and Piehler [24]	Evaluate if ML can exceed expert-level SPE interpretation.	Feature-based classifiers and CNN (Random Forest top).	69,722 SPE samples	RF Accuracy 99.1%, F1 93.2%; outperformed expert panel precision (97% vs. 47%).
Malek, et al. [36]	Rapidly predict M-spike value at point-of-care.	Random Forest regression (43 clinical features).	171 patient records	Excellent agreement (r ≈ 0.95); simplified model needs only prior M-spike and total protein.
Sopasakis, et al. [37]	Compare ML algorithms for automated M-protein identification.	26 tree-based algorithms (Extra Trees, RF, XGBoost, etc).	67,073 CE samples	Top methods reached 97.7% accuracy; iso- type classification was less accurate
Lee, et al. [38]	Classify PEP patterns into diagnostic categories	CNN trained on gel photos and densitogram curves.	2578 images	Per-class AUROC 0.873–0.989; densitograms performed better than raw gel images.
Chen, et al. [39]	Automated SPEP paraprotein screening using frequency domain analysis	Fourier Transform + Spatial Frequency Analysis	1761 total samples	AUC = 0.90; Sensitivity = 0.97; Accuracy = 0.52 (external set)

Note. DL: Deep Learning, ML: Machine Learning, AUROC: Area Under the Receiver Operating Curve, CNN: Convoluted Neural Network, RF: Random Forest, XGBoost: Extreme Gradient Boosting, PEP: Protein Electrophoresis, SPEP: Serum Protein Electrophoresis, CE: Capillary Electrophoresis.

**Table 4:** Future directions and gaps for IFE and SPE studies.

Thematic Gap	Modality	Study	Identified Future Directions
Explainability and Trust	IFE	Wei, Yang, Zhang, Liao, Sheng, Zhou, Wu and Du [28]	Quantify interpretability; expand to oligo/bi-clonal patterns.
	IFE	Thiemann, Klitzke, Martinetz, Gruening, Käster, Barth, Kramer and Martinetz [30]	Add explainability overlays; reduce inconclusive cases via ensemble models.
	SPE	Elfert, Kaminski, Matek, Hoermann, Axelsen, Marr and Piehler [24]	Emphasise explainable AI; adjust thresholds for specific use cases.
Multimodal Data Fusion	IFE	An, Li and Zhang [29]	Develop multi-modal diagnosis systems combining text and laboratory data.
	SPE	Malek, Wang, Tatsuoka, Cullen, Madabhushi and Driscoll [36]	Incorporate genomics and histology; develop tools for free light chains.
	SPE	Sopasakis, Nilsson, Askenmo, Nyholm, Mattsson Hultén and Rotter Sopasakis [37]	Combine CE data with gel image analysis for better isotype identification; explore anomaly detection.
Standardisation and Generalisation	IFE	Vilarinho Filho, Couto and Souza [31]	Conduct multi-site validation; fuse handcrafted and CNN features; release public datasets.
	IFE	Hu, Xu, Jiang, Cheng, Tao, Liu, Jian, Li and Wang [32]	Quantify domain shift; conduct simulated reader studies; extend to urine IFE.
	SPE	Clavijo, Ryan, Xu and Singh [34]	Adjust instrument algorithms to eliminate bias; work toward standardising measurement.
	SPE	Lee, Jeong, Jeon, Song and Park [38]	Validate on larger external datasets; prioritise densitogram-based analysis.
Clinical Integration & Workflow	SPE	Chabrun, Dieu, Ferre, Gaillard, Mery, Chao de la Barca, Taisne, Urbanski, Reynier and Mirebeau-Prunier [33]	Develop publicly available tools; evaluate potential integration into LIS; extend to immunotyping.
	SPE	Cherkaoui, Penickova and Cotton [35]	Determine probability cutoffs to skip IFE confirmation; broaden to all SPE samples.

Note. IFE: Immunofixation Electrophoresis, SPE: Serum Protein Electrophoresis, CE: Capillary Electrophoresis, CNN: Convoluted Neural Network, LIS: Laboratory Information System.

## Data Availability

No new data were created or analyzed in this study.

## Supplementary Materials

The following supporting information can be downloaded at https://www.mdpi.com/article/10.3390/diagnostics16142201/s1: Table S1: PROBAST+AI Signalling Questions for Model Development; Table S2: PROBAST+AI Signalling Questions for Model Evaluation; Table S3: Overall PROBAST+AI Risk of Bias and Applicability Classifications.

## Abbreviations

The following abbreviations are used in this manuscript:

SPE	Serum protein electrophoresis
IFE	Immunofixation electrophoresis
MM	Multiple Myeloma
MGUS	Monoclonal gammopathy of undetermined significance
FLC	Free light chain
CAP	College of American Pathologists
ML	Machine learning
AI	Artificial intelligence
NLP	Natural language processing
WoS	Web of Science
GMM	Gaussian Mixture Model
DCL	Deep Collocative Learning
ANN	Artificial neural networks
LIS	Laboratory Information Systems
TRIPOD-AI	Transparent Reporting of a Multivariable Prediction Model for Individual Prognosis or Diagnosis
CONSORT-AI	Consolidated Standards of Reporting Trials
