# Supplementary material for: Machine Learning for the Interpretation of Serum Protein and Immunofixation Electrophoresis in Multiple Myeloma: A Scoping Review
Source: Diagnostics (Basel). 2026 Jul 14;16(14):2201. doi: 10.3390/diagnostics16142201 (PMC13409347; doi:10.3390/diagnostics16142201)
Supplement: Supplementary file 1 [file diagnostics-16-02201-s001.zip › diagnostics-4290063-supplementary.pdf]

Supplementary Materials

Table S1. PROBAST+AI signalling questions for model development.

Study/Checklist	1.1	1.2	1.3	2.1	2.2	2.3	2.4	3.1	3.2	3.3	3.4	4.1	4.2	4.3	4.4	4.5
Cherkaoui et al. [35]	Yes	Yes	No	Yes	Yes	Unclear	Yes	Yes	Yes	Unclear	Yes	No	Yes	No	Unclear	Yes
Hu et al. [32]	Yes	Yes	Yes	Yes	Yes	Unclear	Yes	Yes	Yes	No	Yes	Yes	Yes	N/A	Yes	Yes
Thiemann et al. [30]	Yes	Yes	Yes	Yes	Yes	Unclear	Yes	Yes	Yes	No	Yes	Yes	Yes	N/A	No	Yes
Chabrun et al. [33]	Yes	Yes	Yes	Yes	Yes	Unclear	Yes	Yes	Yes	No	Yes	Yes	Yes	N/A	Unclear	Yes
Chen et al. [39]	Yes	Yes	Yes	Yes	Yes	Unclear	Yes	Yes	Yes	No	Yes	No	Yes	N/A	Unclear	Unclear
Clavijo et al. [34]	N/A	N/A	N/A	N/A	N/A	N/A	N/A	N/A	N/A	N/A	N/A	N/A	N/A	N/A	N/A	N/A
Elfert et al. [24]	Yes	Yes	Yes	Yes	Yes	Unclear	Yes	Yes	Yes	Unclear	Yes	Yes	Yes	N/A	Yes	Yes
Malek et al. [36]	Yes	Yes	Yes	Yes	Yes	Unclear	Yes	Yes	Yes	Unclear	Yes	No	Yes	Unclear	Unclear	Yes
Lee et al. [38]	Yes	Yes	Yes	Yes	Yes	Unclear	Yes	Yes	Yes	No	Yes	Yes	Yes	N/A	Unclear	No
Sopasakis et al. [37]	Yes	Yes	Yes	Yes	Yes	Unclear	Yes	Yes	Yes	No	Yes	Yes	Yes	N/A	No	Yes
Vilarinho Filho et al. [31]	Yes	Yes	No	Yes	Yes	Unclear	Yes	Yes	Yes	Unclear	Yes	No	Yes	N/A	Unclear	Yes
Wei et al. [28]	Yes	Yes	Yes	Yes	Yes	Unclear	Yes	Yes	Yes	No	Yes	Yes	Yes	N/A	Yes	Yes
An et al. [29]	Yes	Yes	Yes	Yes	Yes	Unclear	Yes	Yes	Yes	Unclear	Yes	Yes	Yes	N/A	Yes	Yes

Table S2. PROBAST+AI signalling questions for model evaluation.

Study/Checklist	1.1	1.2	1.3	2.1	2.2	2.3	2.4	3.1	3.2	3.3	3.4	4.1	4.2	4.3	4.4	4.5	4.6	4.7
Cherkaoui et al. [35]	Yes	Yes	No	Yes	Yes	Unclear	Yes	Yes	Yes	Unclear	Yes	Yes	No	No	Unclear	Yes	Yes	Yes
Hu et al. [32]	Yes	Yes	Yes	Yes	Yes	Unclear	Yes	Yes	Yes	No	Yes	Yes	Yes	N/A	Yes	Yes	Yes	Yes
Thiemann et al. [30]	Yes	Yes	Yes	Yes	Yes	Unclear	Yes	Yes	Yes	No	Yes	Yes	Yes	N/A	No	Yes	Yes	Yes
Chabrun et al. [33]	Yes	Yes	Yes	Yes	Yes	Unclear	Yes	Yes	Yes	No	Yes	Yes	Yes	N/A	Unclear	Yes	Yes	Yes
Chen et al. [39]	Yes	Yes	Yes	Yes	Yes	Unclear	Yes	Yes	Yes	No	Yes	Yes	No	N/A	Unclear	Yes	N/A	Yes
Clavijo et al. [34]	N/A	N/A	N/A	N/A	N/A	N/A	N/A	N/A	N/A	N/A	N/A	N/A	N/A	N/A	N/A	N/A	N/A	N/A
Elfert et al. [24]	Yes	Yes	Yes	Yes	Yes	Unclear	Yes	Yes	Yes	Unclear	Yes	Yes	Yes	N/A	Yes	Yes	Yes	Yes
Malek et al. [36]	Yes	Yes	Yes	Yes	Yes	Unclear	Yes	Yes	Yes	Unclear	Yes	Yes	No	Unclear	Unclear	Yes	Yes	Yes
Lee et al. [38]	Yes	Yes	Yes	Yes	Yes	Unclear	Yes	Yes	Yes	No	Yes	Yes	Yes	N/A	Unclear	No	Yes	Yes
Sopasakis et al. [37]	Yes	Yes	Yes	Yes	Yes	Unclear	Yes	Yes	Yes	No	Yes	Yes	Yes	N/A	No	Yes	Yes	Yes
Vilarinho Filho et al. [31]	Yes	Yes	No	Yes	Yes	Unclear	Yes	Yes	Yes	Unclear	Yes	Yes	No	N/A	Unclear	Yes	Yes	Yes
Wei et al. [28]	Yes	Yes	Yes	Yes	Yes	Unclear	Yes	Yes	Yes	No	Yes	Yes	Yes	N/A	Yes	Yes	Yes	Yes
An et al. [29]	Yes	Yes	Yes	Yes	Yes	Unclear	Yes	Yes	Yes	Unclear	Yes	Yes	Yes	N/A	Yes	Yes	Yes	Yes

Table S3. Overall PROBAST+AI risk of bias and applicability classifications.

Study	Study Type	Participants & Data	Predictors	Outcomes	Analyses
Cherkaoui et al. [35]	Both	High Risk	Low Risk	Low Risk	High Risk
Hu et al. [32]	Both	Low Risk	Low Risk	Low Risk	Low Risk
Thiemann et al. [30]	Both	Low Risk	Low Risk	Low Risk	Unclear Risk
Chabrun et al. [33]	Both	Low Risk	Low Risk	Low Risk	Low Risk
Chen et al. [39]	Both	Low Risk	Low Risk	Low Risk	High Risk
Clavijo et al. [34]	Both	Low Risk	Low Risk	Low Risk	Low Risk
Elfert et al. [24]	Both	Low Risk	Low Risk	Low Risk	High Risk
Malek et al. [36]	Both	Low Risk	Low Risk	Low Risk	High Risk
Lee et al. [38]	Both	Low Risk	Low Risk	Low Risk	High Risk
Sopasakis et al. [37]	Both	Low Risk	Low Risk	Low Risk	High Risk
Vilarinho Filho et al. [31]	Both	Low Risk	Low Risk	Low Risk	Low Risk
Wei et al. [28]	Both	Low Risk	Low Risk	Low Risk	Low Risk
